# Supplementary material for: Visualizing defect dynamics by assembling the colloidal graphene lattice
Source: Nat Commun. 2023 Mar 18;14:1524. doi: 10.1038/s41467-023-37222-4 (PMC10024684; doi:10.1038/s41467-023-37222-4)
Supplement: Supplementary file 1 — Supplementary Information [file 41467_2023_37222_MOESM1_ESM.pdf]

Supplementary Information for
Visualizing defect dynamics by assembling the colloidal graphene lattice

Swinkels et al

SUPPLEMENTARY NOTES

Supplementary Note 1: Particle size

The effective particle diameter can be determined directly from assembly experiments. We determine the inter-particle distances between particles in the honeycomb lattice. In an assembled structure, the centre-to-centre particle distances of bonded tetramer particles should correspond to twice the particle radius plus twice the patch height plus twice the (short) interaction range. In previous work we have shown that due to the short range of the critical Casimir attraction, this is a suitable measure for determining the effective particle size (i.e. the particle radius plus the patch height), and that very good consistency is observed between this measure and atomic force microscopy (AFM) and scanning electron microscopy (SEM) measurements [1]. This method has the added benefit of being *in situ*; it is not always obvious how parameters determined in AFM/SEM correspond to the particles dispersed in solution. The mean of the inter-particle distance distribution at 2.00µm reflects the effective particle size as estimated from SEM (see Figure 1a in the main text), and a standard deviation of $\sigma = 0.05$µm .

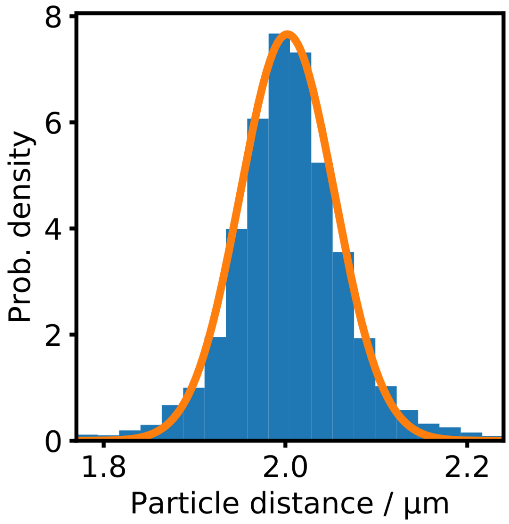

Supplementary Figure 1. **Particle size distribution.** Probability distribution of inter-particle distances between assembled particles. The red line indicates a normal distribution with average particle diameter 2.00µm and standard deviation $\sigma = 0.05$µm. Source data are provided as a Source Data file.

Supplementary Note 2: Diffusion of Particles bound to the surface

To check the influence of the surface attraction on the mobility of the particles, we determined the mean square displacement of particles at the glass at $\Delta T = 0.05$K and $\Delta T = 0.60$K, see Supplementary Fig. 2. These temperatures correspond to the extreme cases of maximal critical Casimir attraction explored, and minimal Casimir attraction required for the particles to adsorb at the surface. Nevertheless, the mean-square displacement of the particles overlap, suggesting negligible influence of the attraction on the mobility: we observe a power-law behaviour with slope ~ 1, as expected for free diffusion, and with diffusion constant $D = 0.88 \mu m^2 s^{-1}$. Furthermore, the two different attractions investigated show the same diffusion constant within error bars. Apparently, the stronger attraction of the particles to the wall does not influence the particles' diffusion through interaction with the wall via hydrodynamic effects or friction.

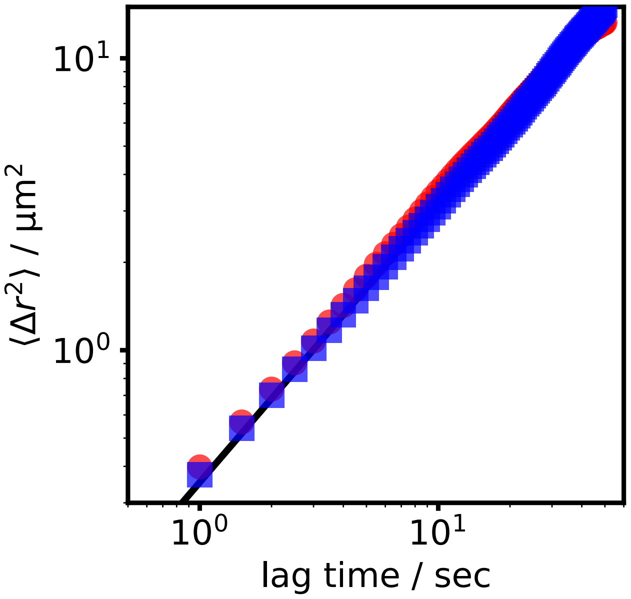

Supplementary Figure 2. **Mean-square displacement (MSD) of particles bound to the surface.** Red circles indicate $\Delta T = 0.60°C$, blue squares $\Delta T = 0.05°C$. The MSD can be fitted using the equation $\langle r^2 \rangle = 4D \cdot t^n$, where D is the diffusion coefficient, t is the lag time, and n is the exponent. In an ideal system of free diffusion, $n = 1$. The black solid line is a fit of our data, yielding a diffusion coefficient $D = 0.88 \mu m^2 s^{-1}$ and $n = 0.95 \pm 0.05$. Source data are provided as a Source Data file.

Supplementary Note 3: Tracking Patchy Particles

We analyse confocal images using particle tracking software (Trackpy [2]) to determine the centre of each fluorescent patch. In our experiments, the particles are all at the same height, having one patch bound to the capillary surface and the other three available for bonding. In Supplementary Figure 3a, we show a typical confocal microscope image of the tetrapatch particles bonded in a 6-membered ring (hexagon), and in Supplementary Figure 3b we show a schematic reconstruction with the particle patches indicated in orange. In this reconstruction, each particle has one patch in the centre of the particle, and 3 patches at the boundary. To find particle positions, we perform the following actions:

1. Determine the location of each individual fluorescent patch using Trackpy in 2 dimensions (the z-axis is fixed in this system, so determination is possible, but superfluous).

2. Find all other patches within approximately one particle diameter using a kd-tree (implemented in SciPy), and connect those into a network.

3. In the resulting network (Supplementary Figure 3c), we find all triangles.

4. For each triangle, we determine the mean edge length and the variation of this value. We only select triangles where the mean edge length d is close to the expected value based on radius r ($d = \frac{4}{\sqrt{3}}r$), and where the edge length variation is small, see Supplementary Fig. 3d and e. This means we effectively select the blue triangles in Supplementary Fig. 3c.

We are left with a triangle with 3 vertices corresponding to the outer patches. In the centre of the triangle is the patch bound to the surface. Based on this procedure, bonds between particles are then easily determined by checking which patches are shared between particles.

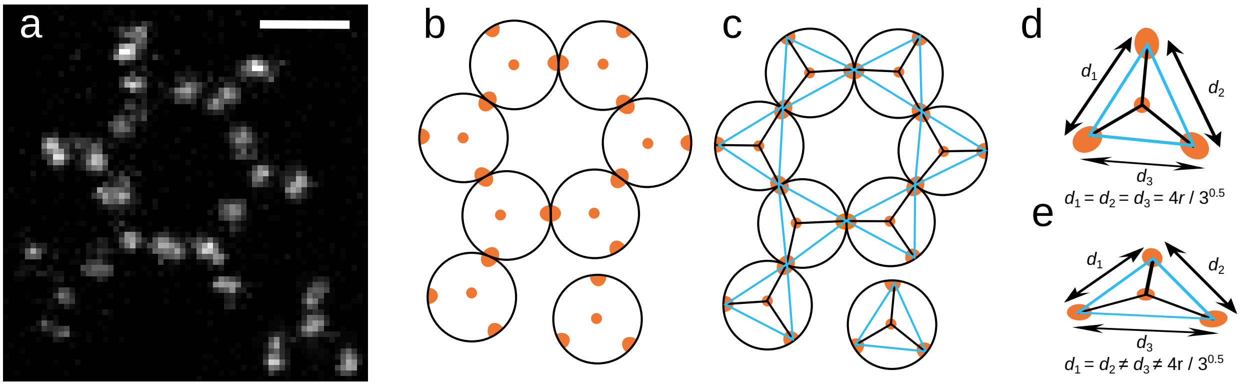

Supplementary Figure 3. Tracking and reconstruction procedure. (a) Representative z-projected confocal microscope image of tetrapatch particles bonded into a hexagon. Scale bar indicates 5µm. (b) Schematic reconstruction of the particle arrangement corresponding to (a). Particles have a black outline, patches are shown in orange. In (a), the central patch is slightly out of focus, and is thus less bright. (c) Corresponding network formed by connecting all patches within a particle radius of each other. Regular triangles shown in cyan demarcate the connected outer patches belonging to one particle. (d) A group of 4 patches belonging to the same particle, forming a regular tetrahedron, which has edges with the expected length. All edges have equal lengths within particle dispersity and tracking errors. (e) A group of 4 patches not belonging to the same particle, forming an irregular tetrahedron, where not all edges have the same length, and not all edges have the expected length.

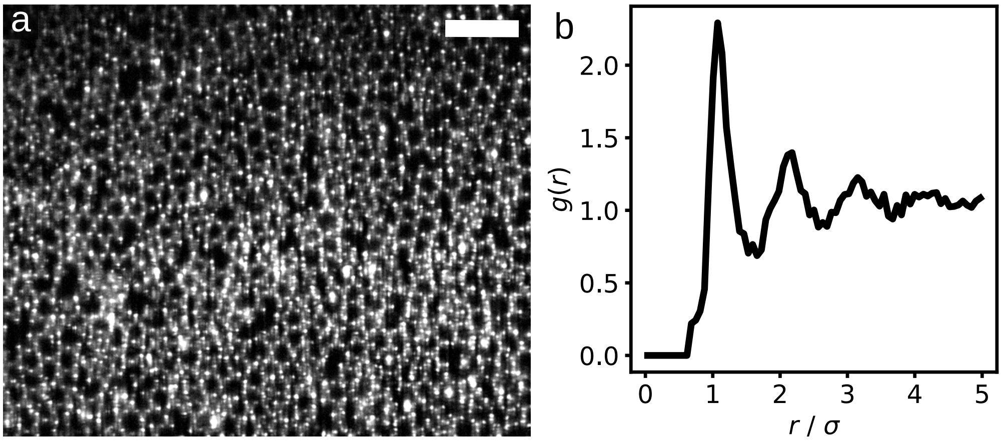

Supplementary Figure 4. **Amorphous network.** When our system is quickly quenched to high attractive strength, it arrests in an amorphous network state. (a) A confocal microscope image of such a state and (b) its accompanying radial distribution function. Source data are provided as a Source Data file. Scale bar indicates 10µm.

Supplementary Note 4: Fast quenching to large attractive strength

Normally, we slowly increase the temperature of the system in small, slow steps, waiting at each temperature for four hours, to ensure we observe equilibrium conditions. When we do not do this, and instead heat the system from completely unattractive ($\Delta T = 0.40°$C) to strongly attractive ($\Delta T = 0.05°$C), we obtain an amorphous structure, see Supplementary Fig. 4. The formation of such an amorphous structure is in line with our expectations: a very similar process occurs in any other colloidal assembly process, and indeed in atomic systems as well when a fast quench is applied.

Supplementary Fig. 4 shows an interesting amorphous structure; although there is no clear order, the structure is still very open, and forms rings. As such, there is some local order, even if it disappears at long range.

Supplementary Note 5: Patchy Particle Assembly

To assemble the particles near-equilibrium, we slowly increase the temperature in steps of 0.05°C starting from $\Delta T = 0.25$°C, waiting at each temperature for 4 hours. This way, the system has time to adjust to the new attractive strength and acquire a near-equilibrium state. In Supplementary Fig. 5, we show the typical process of assembly. A bright-field microscope image of the system at a low density after approx. 17 hours of assembly is shown in Supplementary Fig. 5a.

To follow network formation, we perform a ramp (increasing temperature by 0.05°C every 2.6 hour) and track the particles as assembly progresses. The size of the largest cluster as a function of time is shown in Fig. 5b. Clearly, at every temperature jump, the cluster size grows rapidly, and subsequently plateaus at an equilibrium cluster size. At $\Delta T = 0.25$°C, the largest cluster only consists of approx. 10 particles, while after temperature increase to $\Delta T = 0.10$°C, we obtain large clusters of over a thousand particles, containing practically all particles in the field of view.

We compare the relative amounts of ring motifs in Fig. 5c. Initially, there are no, or very few rings. Starting from $\Delta T = 0.20$°C, pentagons, hexagons and heptagons start to form, with the majority being pentagons. As the attraction increases (ΔT decreases), the number of 5-membered rings drops compared to the number of hexagons. At $\Delta T = 0.10$°C, hexagons become more common than pentagons. The fraction of 7-membered rings remains closely constant over time and temperature.

To study the formation of the patchy particle network in more detail, we determine the equilibrium cluster mass distribution at different ΔT, as shown in Supplementary Fig. 6a. At low attraction, the distribution is cut off at small cluster mass. As the attraction increases (ΔT decreases), the mass cut-off moves to the right, indicating formation of larger structures, and eventually, at $\Delta T = 0.1$°C, the distribution assumes a power law, indicating percolation of the assembled structure across the plane. This behaviour is qualitatively in line with what we expect for a growing network of patchy particles [3].

We also determine the diameter of the assembled structures, and show a contour plot of diameter frequency as a function of mass in Supplementary Fig. 6b. Initially, at low cluster mass, the diameter increases with a power of 1, indicating growth of linear structures. At higher mass, the diameter increases with the lower power of 1/2, indicating the growth of

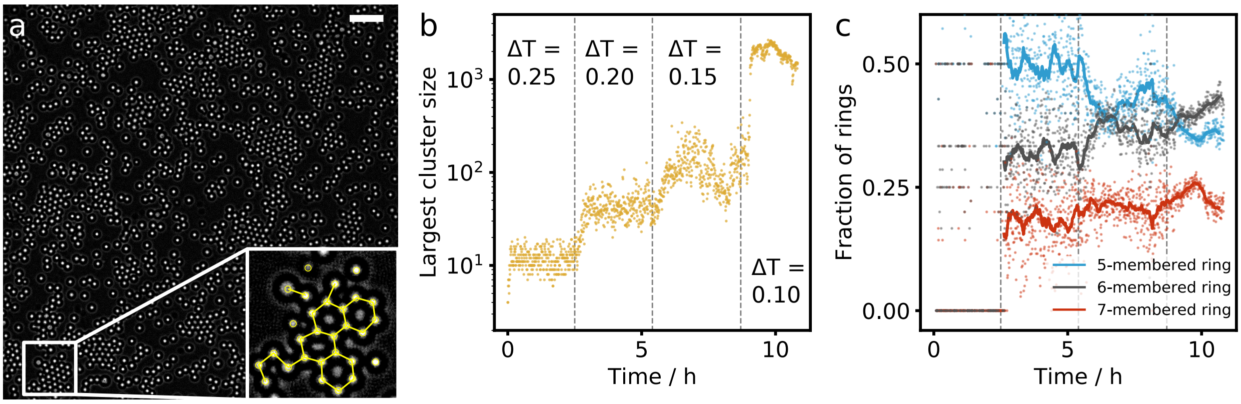

Supplementary Figure 5. **Assembly of patchy particles.** We assemble particles by performing a step-wise temperature ramp: we start at $T = 33.70°C$ ($\Delta T = 0.25°C$). Every 4 hours, we increase the temperature (equivalent to decreasing ΔT) by 0.05°C. (a) Bright field microscopy image of assembled particles after approx. 17 hours, at $\Delta T = 0.05°C$. Most particles are part of a small structure. The inset shows that particles are ordered hexagonally. Scale bar indicates 10µm. (b) We perform a slightly faster ramp to follow the size of the largest cluster as a function of time. Dashed vertical lines indicate temperature changes. (c) During this ramp, we track the fraction of rings that are pentagons (blue), hexagons (grey), and heptagons (red) as a function of time. Dots show data for individual frames, and solid lines show the moving average over 10 minutes (20 datapoints). Initially, there are no/very few rings, but as ΔT becomes smaller, larger structures, including rings are formed. At $\Delta T = 0.20°C$, the majority of rings is pentagons. However, with decreasing ΔT, hexagonal rings take over. Source data are provided as a Source Data file.

two-dimensional structures.

The transition to the power-law slope 1/2 is thus related to the transition from chains to closed clusters: for masses below 4, the cluster grows as a linear chain, whose length grows linearly as particles are added. When the clusters grow larger, 2D morphologies become available, like rings, branched chains, and eventually the colloidal graphene lattice. This leads to a transition towards power-law slope of 2, consistent with regular 2D growth. Interestingly, this general scenario is robust, and the evolution of the diameter-mass distribution is largely independent of the density, attraction, and other experimental details of the experiment.

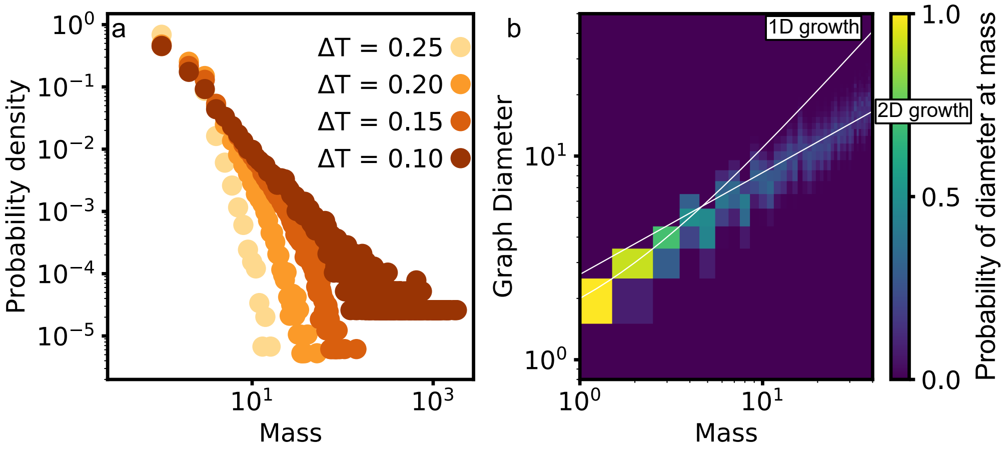

Supplementary Figure 6. **Networking.** (a) Cluster mass distribution of a structure at four different ΔT. As we increase the attractive strength between patches (darker colours), the clusters that are formed are typically larger. At the smallest ΔT, the cluster-mass distribution adopts a power law, indicating a plane-spanning structure. (b) Probability of finding a graph diameter as a function of mass. Yellow indicates high probability, blue indicates low probability. Initially, clusters grow linearly, resulting in growth with power 1. As 2-dimensional morphologies become available, 2D growth dominates and the power decreases to 1/2.

Supplementary Note 6: Radial and Bending potential of a patchy particle

The critical Casimir potential is short ranged, its magnitude and range are set by the temperature offset $\Delta T = T - T_\mathrm{c}$ to the critical temperature, T_c. Further factors that determine the magnitude of the potential are the absorption preference of the surfaces (particle patches, glass surface), and the composition of the binary solvent, as detailed in [4]. By formulating the critical Casimir potential model presented in [4] for patchy particles and benchmarking it onto our patchy particles as shown in [5], we arrive at the attractive potentials shown in Supplementary Fig. 7a. These potentials are valid for ideally opposing patches (no bond bending strain), which is not the case here. Due to the glass-bound patch fixed vertically and the tetragonal patch arrangement, there is always a bonding angle of approx. 60° between the patches in the vertical plane (see Figure 1C in the main text). Since this bonding angle is the same for each particle, it equally reduces the bond energy between all particles. Our experiments mostly take place at $\Delta T = 0.05$°C, which means the bonding energy of particles is approximately $15k_\mathrm{B}T$.

The bond-bending potential is harder to estimate theoretically, but can be determined from experimental measurements. We follow five separate clusters of 3 particles, and track the fluctuations of the internal bonding angle of these particles (see inset of Fig. 1d of the main text). A histogram of angle deviations from the ideal bonding angle is shown in Supplementary Fig. 7b. If we assume the angle distribution is purely due to equilibrium fluctuations, they follow a Boltzmann distribution of the form $P(\theta) = P_0 e^{-U_{bend}(\theta)/k_\mathrm{B}T}$. We can thus convert our measured probabilities to a bending energy distribution using $U_{bend}(\theta)/k_\mathrm{B}T = ln(P(\theta)) + c$, where we choose the arbitrary energy offset c such that $U(0) = 0$. The resulting bending energy distribution is shown in Fig. 7c. Finally, we fit the potential with a parabola assuming a harmonic potential (Hooke's law), shown as a black solid line in Supplementary Fig. 7c. This leads to a bending stiffness with a force constant of $39.4k_\mathrm{B}T/rad^2$. We also plot the same fit in Supplementary Fig. 7b, by converting values via the Boltzmann distribution, showing that in both cases, the simple harmonic fit describes our data well.

With this bending stiffness, we can now also determine the energy associated with the bending strain of different n-membered rings (n-gons), assuming equal distortion of all bond angles, as shown in Supplementary Fig. 7d (and Fig. 1d of the main text). As expected,

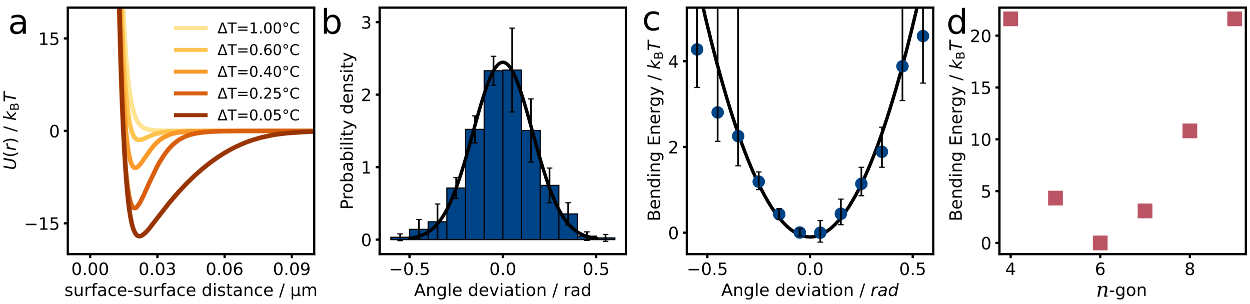

Supplementary Figure 7. Bond energies of Patchy Particles (a) Radial patch-patch interaction potential estimated by modelled data benchmarked onto experiments [5]. (b) Distribution of the bond angle between two attached particles, bonded to a central one. We track 5 independent three-particle clusters for 100-200 frames, at a frame rate of 1 fps (all at $\Delta T = 0.05°C$). The error bars show the standard deviation between the 5 measurements. (c) The bending potential between the 3 patchy particles as determined from (b). The black solid line is a Hookean fit, which assumes a simple harmonic bending potential $U_{\text{bend}} = \frac{1}{2}k_{\text{bend}}\theta^2$, with force constant $k_{\text{bend}} = 39.4k_{\text{B}}T/\text{rad}^2 = 0.012k_{\text{B}}T/(°)^2$ as mentioned in the main text. The error bars are carried over from panel b. (d) Total bending energy of a regular n-membered ring, assuming the force constant determined in panel (c), and that all bonds are bend equally. Source data are provided as a Source Data file.

the bending energy vanishes for hexagons whose inner bond angles match that of the patchy particle. Rings with more or less particles, however, exhibit significant bond bending energy as the inner bond angle deviates from the ideal one. For pentagons and heptagons, the bending energy is only between 4 and $5k_{\text{B}}T$; thus, despite the bending energy cost, pentagons and heptagons are likely to form, which is indeed what we observe.

Supplementary Note 7: Comparison of defects in atomic and colloidal graphene

As discussed in the main text, not all defects found in atomic graphene are present in the colloidal graphene lattice. In this section, we look at a few common defects in atomic graphene, and discuss how their energies compare to the colloidal analogue, based on the force constants determined in Supplementary Note 6. We start by taking the known topology of atomic graphene defects, and scaling it up to the micrometer scale of the colloidal graphene lattice such that the equilibrium C-C bond distance is mapped onto the equilibrium distance in our colloidal lattice (from 1.43 Å to 2µm, an approximately $1.4 \cdot 10^5$ times increase in scale) [6, 7]. We can then calculate the energy of the structure as a result of bond stretching and bond bending, using the potentials shown in Supplementary Figure 7. Although this method will only yield approximate energies, it will help illustrate why certain defects are not present in colloidal graphene where we might expect them.

The Stone-Wales defects

The Stone-Wales defect is a very common type of defect in graphene. It is essentially caused by a particle pair turning 90°, leading to the formation of two 7-membered and two 5-membered rings, as shown schematically in Supplementary Fig. 8 (defect a). The defect is never observed in the colloidal graphene lattice. Using the bending and radial potentials determined from Supplementary Note 6, we can estimate the energies of formation of the Stone Wales defect geometry [6, 7] in colloidal graphene. We find that while the formation of 2 heptagons and 2 pentagons causes only a moderate bending energy cost (approx. $24k_{\mathrm{B}}T$ in total), small changes in the inter-particle distances lead to much more prominent radial potential energy costs. In atomic graphene, the defect induces a compression of several atomic bonds by up to 3% [6, 7]. Such compression is incompatible with the steep, short-ranged screened electrostatic and critical Casimir potentials: Supplementary Figure 7a shows that a compression of just 20nm (about 1% of the particle-particle bond length) from the ideal bond length results in a huge energy penalty, much larger than the vertical scale of the figure. Therefore, it will be energetically highly unfavourable to accommodate a Stone-Wales defect in colloidal graphene, and it will in practice never occur, consistent with our observations.

We note that this does not entirely exclude the formation of a Stone-Wales-like defect in the colloidal graphene lattice: a different configuration that does not compress bonds may still exist. Yet, finding such a configuration is beyond the scope of the current work.

The case of the Stone-Wales defect is representative of other common defects in atomic graphene. The geometry of these defects as observed in the atomic graphene lattice often involves bond compressions, because a compression carries only moderate energy penalty. The compressions are then balanced against bending and stretching energies. In the colloidal case investigated here, the amount of bond compression will be minimal compared to bond bending (and to some extent also to bond stretching, see the asymmetric shape of the radial potential in Supplementary Figure 7a) due to its very high energy cost.

Single vacancies

Single vacancies are generally understood to exist in graphene in two distinct ways, the symmetric monovacancy and the 59 (or 'reconfigured') monovacancy, shown in Supplementary Fig. 8 (defects b and c) [8]. In atomic graphene, a reconfigured vacancy has only a marginally smaller energy compared to the symmetric one (smaller by only 0.2eV [9]), so there is a small driving force for reconfiguration. This is because the lack of dangling bonds in the 59 defect is favourable compared to the symmetric defect, but is mostly balanced by the energy penalty due to deformation of the crystal lattice around the 59 vacancy [9].

Like in the atomic case, in the colloidal case, the formation of a symmetric or a 59 monovacancy depends on the balance of the energy of dangling bonds against that of reconfiguration of the lattice. However, in the colloidal case, significant bond compression leads to a very large energy penalty, making it unfavourable, just like the Stone-Wales defects. The 59 defect like it is observed in the atomic lattice can therefore not be formed in the colloidal graphene lattice.

However, a non-compressed conformation of the 59 monovacancy is possible to assemble, although at the cost of additional bond bending, as can be intuitively explored with a simple ball-and-stick molecule building set. This additional bending in the non-compressed 59 defect variant will likely induce significant additional bending energy, explaining why we do not observe it in colloidal graphene.

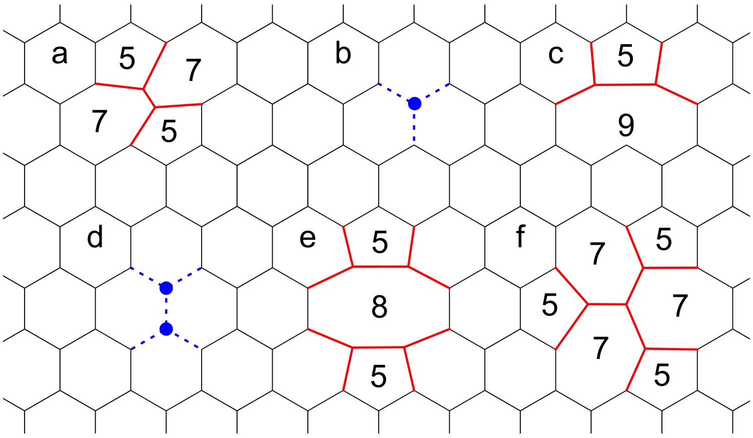

Supplementary Figure 8. **Schematic structure of several defects in the honeycomb lattice.** The different defect types are labelled a-f in the lattice, showing a) a Stone-Wales defect, caused by a particle pair rotating $90°C$, resulting in two 5-membered rings and two 7-membered rings. b) a symmetric single vacancy. One particle is missing from the lattice, which means there are 3 dangling bonds. c) a monovacancy reconfigured into a 59 defect. This reconfiguration leaves only 1 dangling bond, but does have stretched bonds and strained bond angles. d) a symmetric divacancy. Two particles are missing from the lattice, resulting in four dangling bonds. e) a divacancy reconfigured into a 585 defect. This results in the elimination of all dangling bonds, in exchange for strained bond angles and stretched bonds. f) a divacancy reconfigured into a 555-777 defect. Like the 585 defect, all dangling bonds are removed, but in exchange for strained bond angles and stretched bonds. Red lines indicate strongly deformed bonds, blue dots and dashed lines indicate missing particles and bonds.

Double vacancy

Double vacancies exist in the honeycomb lattice in three configurations, the symmetric divacancy (Supplementary Fig. 8, defect d), the 585 divacancy (Supplementary Fig. 8, defect e), and the 555-777 divacancy (Supplementary Fig. 8, defect f). Both the 555-777 and the 585 defects have significantly lower energies compared to the symmetric divacancy in the atomic case, reflected in their ubiquity in defected graphene [10].

However, in the colloidal case, we never observe these vacancy reconfigurations. We estimate the corresponding bending energy contributions to be $29.5 k_{\mathrm{B}}T$ (for the 555-777 divacancy) and $34.4 k_{\mathrm{B}}T$ (for the 585 divacancy). While these are relatively moderate, again

the incompressibility of the particles causes an exceedingly high energy penalty. Furthermore, the high activation energy of such a reconfiguration makes the formation of these defects unlikely.

In general, larger vacancies in colloidal graphene have a lower energy penalty for reconfiguration to a state with fewer or no dangling bonds. Accordingly, activation energy barriers are easier to overcome for larger vacancies. Therefore, unlike small vacancies, large vacancies in colloidal graphene can reconfigure to a lower energy state, as we indeed observe in the yellow region of Figure 4a (main text) for example.

Supplementary Note 8: Energy of ring formation

To illustrate the kinetic pathway that leads to the formation of pentagons rather than the equilibrium hexagon motif, in the manuscript we show energy traces of particle clusters as they close into pentagons and hexagons (Fig. 3d and e of the main text). To obtain the configurational energies shown, we have tracked the particles as usual and determined the total energy of the cluster from the particles' bond angles and saturation of bonds. Hence, the energy is determined in the same way as in Figure 4d of the main text, that is, we have used the sum of the bending energies (using the force constant determined in Supplementary Fig. 7) and the dangling bond energies of the particles involved in the assembly. For convenience, the energy of the ideal 6-membered ring was set to $0k_{\mathrm{B}}T$ as reference point.

To facilitate comparison, the resulting expected energy levels of five different structures that we observe in the movies are indicated: 3 particles bonded in an open chain (labelled I), 4 particles bonded in an open chain (II), 5 particles bonded in an open chain (III), and closed rings of 5 particles (pentagon, red) and 6 particles (hexagon, blue).

Initially, the three bonded particles have a total energy of approximately $62k_{\mathrm{B}}T$ (structure I). The observed small energy fluctuations are the result of bond-bending due to thermal fluctuations. After 8-minutes, first a fourth particle, and subsequently a fifth particle binds, after which the ring almost immediately closes into a pentagon. The energy drops via the corresponding intermediate values to $\sim 23k_{\mathrm{B}}T$, corresponding to the formation of the pentagon. The process is fast, and the intermediate states II and III are only observed for a single frame each. After the energy drop, the pentagonal ring opens and closes a few times to go back to the open structure III; however, since no sixth particle is in the correct position to bind, the ring closes before a sixth particle can be incorporated. This way, the pentagon remains metastable.

In the case of hexagon formation, the observation starts with structure II. Now, when the fifth particle binds after 15min, the structure remains in the open state (III); at this point, either a pentagon or a hexagon can form, as indicated in panel e (red and blue arrow, respectively). This time, another particle is available and binds, and the ring closes into a hexagon after 20min. This is accompanied by a significant drop in energy to the ground state.

The two examples illustrate in detail the energetic pathway that groups of particles can take to form a ring structure, and are reminiscent of molecular equilibrium reactions. Initially, when structure III is formed, it is very likely that a pentagon forms shortly after, because this path is energetically favourable though it does not lead to the energetic ground state. Formation of the hexagon requires a 6th particle to be available for bonding. Once a pentagon has formed, an activation energy barrier $E_{\mathrm{act}} \sim 10 k_{\mathrm{B}} T$ needs to be overcome to open the ring so that another attempt at reacting to a hexagon can occur. If no particle is available, the ring will close again. Several of these attempts are clearly seen in the energy trace of the pentagon (red) in panel (d), where the energy jumps to the level of the open ring, but immediately drops down again into that of the closed ring. In this case of an isolated ring, such attempts occur frequently. However, when the ring is embedded in a larger structure, E_{act} can become much bigger due to multiple bonds that need to be broken, to an extent where it is virtually impossible, and the system freezes.

Supplementary Note 9: Merging of grains

When multiple crystal grains meet during assembly, they will attempt to merge into one bigger grain to minimize the number of dangling bonds. If the two crystals are aligned, the two crystals can be merged easily, resulting in one bigger crystal with no defect. This seamless merging has been mentioned in the main text, and we provide an example in Supplementary Fig. 9. Four different crystals meet and form one larger grain. The red and blue grains merge, and form a new 6-membered ring at their interface. The combination of yellow, green and blue also yield 6-membered rings. The yellow and green clusters are both small structures with no or a few closed rings, and are therefore more flexible with regard to their orientation. The creation of a complete 6-membered ring is therefore more likely.

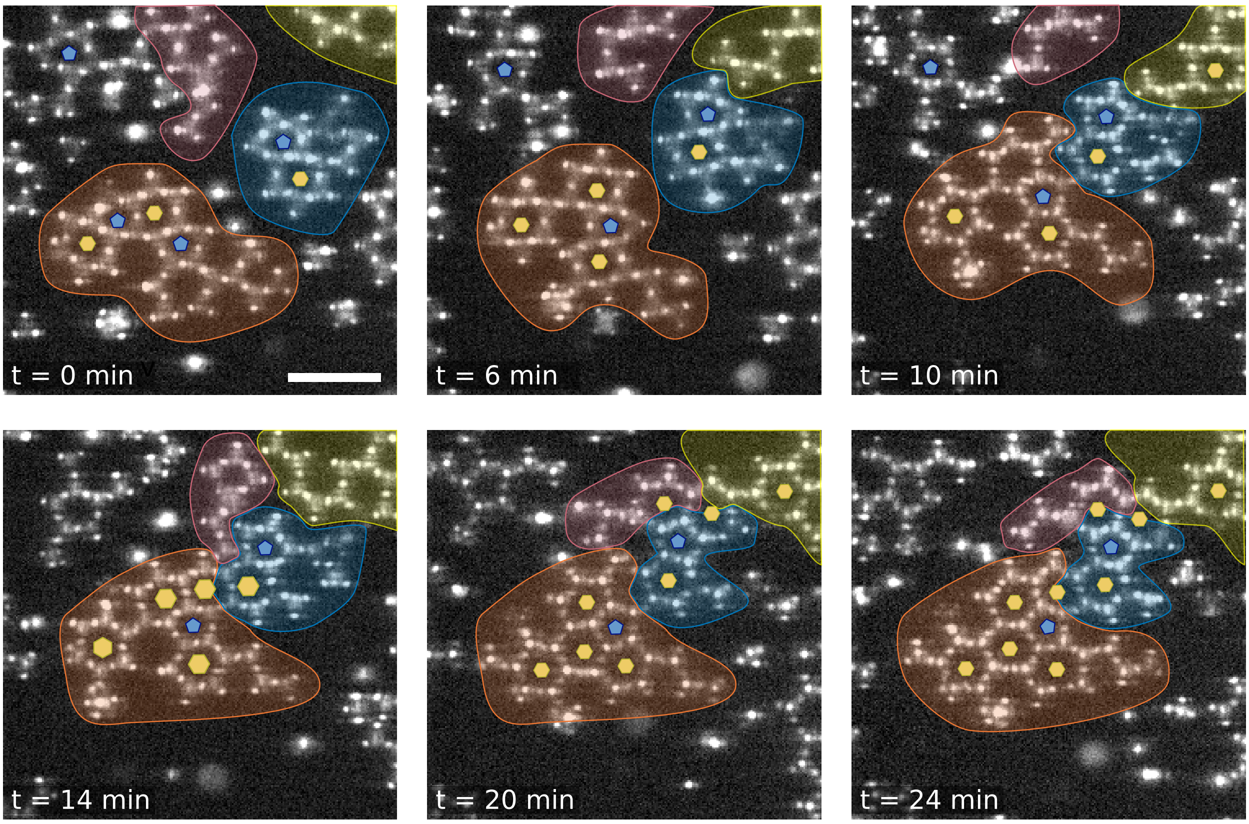

Supplementary Figure 9. **Four crystal grains merge into one.** Confocal snapshots of the seamless merging of 4 crystal grains into one. The grains, fully separated at $t = 0$ min, are colour coded to facilitate tracking. Yellow symbols indicate hexagons, blue symbols indicate pentagons. Scale bar indicates 5µm.

Supplementary Note 10: Building a defect-free lattice

One might wonder if it is possible to create a perfect monocrystal of honeycomb lattice, without defects. In this respect, our system relates to the diamond lattice: the kinetic traps we observe in our experiments mirror those encountered in assembling colloidal diamond. In both cases, the system generates kinetically favoured 5-membered rings, while 6-membered rings are the equilibrium motif of the formation of the honeycomb or diamond lattice [11].

In a recent publication about the experimental assembly of colloidal diamond the "pentagon problem" was avoided by shaping the constituent colloids to make the formation of 5-membered rings highly unfavourable [12]. A similar strategy could be employed in our system: particles could be designed specifically to not be able to form non-hexagons. This prospect poses a challenge synthesis-wise, but with increasing control over patchy particle synthesis, this may be feasible in the future.

Another interesting strategy for suppressing pentagon formation in colloidal diamond was recently proposed by Neophytou et al. [13] in simulations: the authors suggest selectively forming only even-numbered rings. By dividing particles into two types that can mutually bond to each other, but not to particles of the same type, 5- and 7-membered rings are suppressed, leading to the formation of only hexagons (4- and 8- membered rings are energetically unfavourable). This strategy would surely work in our honeycomb-lattice system as well. Unfortunately, creating orthogonally binding A-B particles is not easily realized in our critical Casimir interaction-based system due to the universal nature of attractions: each patch will attract any other patch. Other systems may be more successful here; orthogonally assembling DNA-coated colloids are relatively simple to make, and may be a route to creating a defectless colloidal honeycomb lattice [14].

Furthermore, more traditional annealing methods to create defect-free crystal lattices can be applied directly to our system, as we can reversibly change the strength of the critical Casimir interaction. Careful annealing via specific routes, with heating and cooling cycles, could in principle eliminate defects, a strategy also often employed for defect-free atomic graphene [15]. We have repeatedly attempted different annealing protocols, but with limited success. We hypothesize that the energy difference between the defected and regular state is too small, so the required temperature changes are smaller than the resolution of our heating setup (in the order of $0.01K$). This makes purposeful annealing ineffective in our

setup. This is a technical limitation, while the process may be possible in principle.

Secondly, to prevent defects, one could introduce a small nucleation point, from which the lattice can grow, instead of letting regular nucleation-and-growth dominate the assembly process, leading to polycrystals. This method is also employed in attempts at generating defect-free atomic graphene [15]. Our initial efforts, however, were similarly ineffective. Again, technical limitations may have played a role: for successful growth, one needs to bring the sample very close to the nucleation temperature, and then introduce a very small point, where the attraction is slightly higher (i.e. the binary solvent temperature closer to T_c). Nevertheless, it is experimentally challenging to achieve such precision, as the temperature is already close to T_c. Furthermore, 5-membered rings may still form on the edge of the growing lattice, and these can still be stabilized by forming a pentagon-heptagon pair.

Finally, the usage of a template can be considered as a possible way to realize a defect-free lattice. This strategy should work well with our patchy particles that are already attached to the surface with one of their patches. Using a surface with specifically arranged attractive (hydrophobic) spots, the particles could be forced into a perfect honeycomb lattice. In this case, the surface patterning would even fix the lattice orientation, which would make the stitching of lattices less defect-prone [16]. This strategy is technologically feasible with modern surface patterning techniques, and could be used to create well-aligned, well-ordered honeycomb lattices. Such template-directed assembly would however no longer allow the study of free honeycomb assembly in analogy to atomic graphene, which is what we have aimed at in the current paper.

Supplementary Note 11: Particle dynamics in the polycrystal

In Figure 4 of the main text, we investigate the difference in dynamics of two regions of a polycrystal: one relatively static region (red) around a 5-7 grain boundary, and one relatively dynamic region (yellow), around a poly-vacancy. Apart from the measures we give in Figure 4d-f of the main text, which rely on static particle configurations, we can also look at the motion of the particles more directly to obtain insight into the dynamics.

Unfortunately, direct particle tracking between frames is difficult due to the long time interval of 9 hours and photo bleaching of the dye over many images. We therefore resort to the raw images. To extract the dynamics, we first stabilize the view on the lattice, removing drift and other collective movement from the data (using the ImageJ StackReg plugin [17]). We then calculate the standard deviation and mean intensity of each pixel over the entire measurement period in the field of view. The ratio between the standard deviation and mean intensity $K = \sigma/\langle I \rangle$ can be used as a measure of the dynamics at every point in the sample. Here, the standard deviation is calculated over all recorded images in the 9-hour interval. Confocal microscopy images of the red and yellow regions of Fig. 4 of the main text are depicted in Supplementary Fig. 10a and b. On the right, we show a colour-coded map of K of the areas highlighted in panels a and b. In these images, low values (blue) indicate little change in intensity, meaning a static structure, while high values (yellow) indicate strong intensity fluctuations, and thus a dynamic structure. Clearly, some high-mobility regions are observed in the yellow region, indicating particles that have spent time bonding and de-bonding. At the same time, some longer-lived (but still transient) structures can be distinguished in the central region from a darker colour (dotted red line guiding the eye).

It is unfortunately not straightforward to perform this analysis on the entire polycrystal. In smaller regions that have matching translational and rotational drift, movement can be accounted for using standard image processing methods as described above. In the full poly-lattice however, individual crystals drift relative to each other on experimental timescales, making this analysis prohibitively difficult.

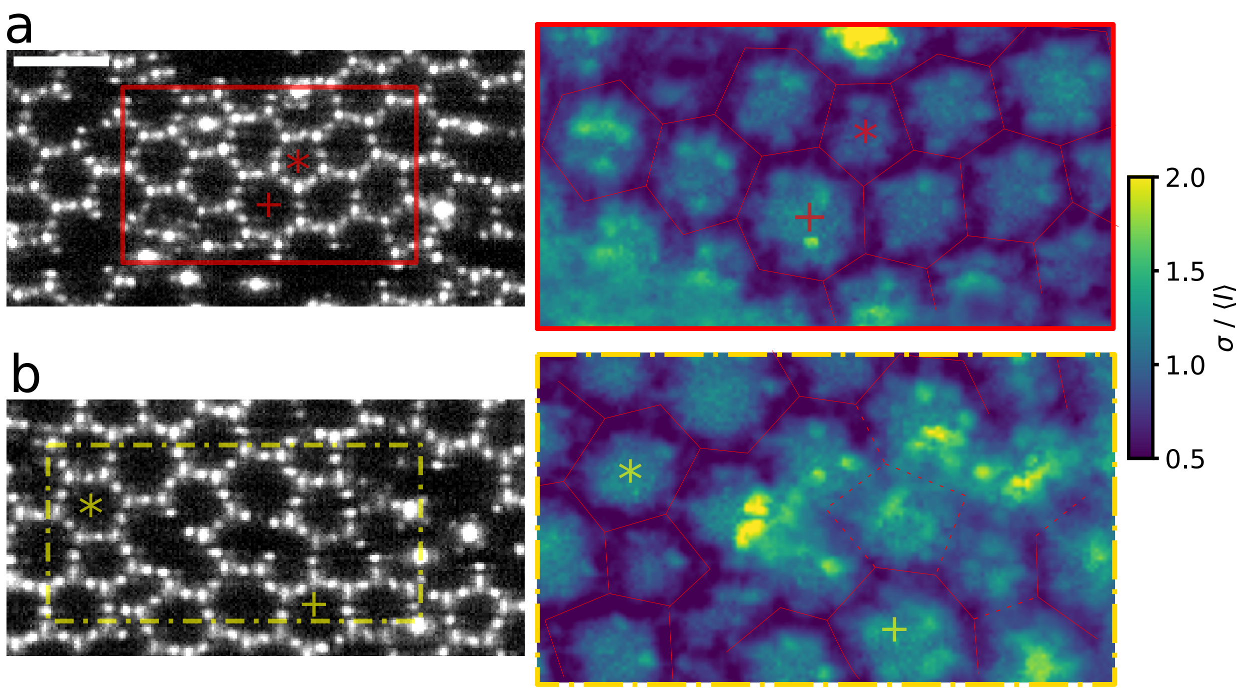

Supplementary Figure 10. **Dynamics of static and dynamic regions in a polycrystal.** (a, b) Confocal microscope images of the red (static) and yellow (dynamic) regions of Fig. 4 of the main text. The regions highlighted in red/yellow on the left are shown on the right, colour coded for $K = \sigma/\langle I \rangle$. Here, blue indicates low K, and thus static structures, while yellow indicates high K, and thus dynamic structures. The red symbols and dashed lines are guides to the eye, to link between the images on the left and right. Scale bar indicates 5µm.

SUPPLEMENTARY METHODS

Supplementary Method 1: Sample Preparation and Glass Silanization

The patchy particles are stored dispersed in water with a small amount of F108 copolymer as stabilizer ($< 0.05\%$wt). To prepare a sample stock, particles are transferred to the binary solvent by washing them at least 4 times in the desired binary mixture. To prepare a sample for microscopy, a small amount of the sample is injected into a glass capillary (Vitrotubes, Rectangle Boro Tubing 0.20×2.00mm) and sealed with teflon grease (Krytox GPL-205).

The glass capillaries are silanized, making them hydrophobic. The hydrophobic treatment for capillaries is a simple gas silanization reaction, briefly: The capillaries are cleaned thoroughly using a piranha treatment, or alternatively using Hellmanex III and plasma treatment. Capillaries are then placed in a vacuum desiccator together with approx. 1 ml of hexamethyldisilazane (HMDS) ($\geq 99.0\%$, Sigma-Aldrich). Pressure is lowered to below 200mbar using a pump, and kept low for at least 2 hours. The capillaries are then baked in an oven at 120°C for circa 1 hour [18–20].

SUPPLEMENTARY REFERENCES

[1] P. J. M. Swinkels, S. G. Stuij, Z. Gong, H. Jonas, N. Ruffino, B. van der Linden, P. G. Bolhuis, S. Sacanna, S. Woutersen, and P. Schall. Revealing pseudorotation and ring-opening reactions in colloidal organic molecules. *Nature Communications*, 12(1):2810, May 2021.

[2] Daniel B. Allan, Thomas Caswell, Nathan C. Keim, Casper M. van der Wel, and Ruben W. Verweij. Soft-matter/trackpy: Trackpy v0.5.0. Zenodo, April 2021.

[3] Francesco Sciortino, Emanuela Bianchi, Jack F. Douglas, and Piero Tartaglia. Self-assembly of patchy particles into polymer chains: A parameter-free comparison between Wertheim theory and Monte Carlo simulation. *Journal of Chemical Physics*, 126(19), 2007.

[4] S. G. Stuij, M. Labbé-Laurent, T. E. Kodger, A. Maciołek, and P. Schall. Critical Casimir interactions between colloids around the critical point of binary solvents. *Soft Matter*, 13(31):5233–5249, 2017.

[5] H. J. Jonas, S. G. Stuij, P. Schall, and P. G. Bolhuis. A temperature-dependent critical Casimir patchy particle model benchmarked onto experiment. *The Journal of Chemical Physics*, 155(3):034902, July 2021.

[6] L. Li, S. Reich, and J. Robertson. Defect energies of graphite: Density-functional calculations. *Physical Review B*, 72(18):184109, November 2005.

[7] Esmaeil Zaminpayma, Mohsen Emami Razavi, and Payman Nayebi. Electronic properties of graphene with single vacancy and Stone-Wales defects. *Applied Surface Science*, 414:101–106, August 2017.

[8] Alex W. Robertson, Barbara Montanari, Kuang He, Christopher S. Allen, Yimin A. Wu, Nicholas M. Harrison, Angus I. Kirkland, and Jamie H. Warner. Structural Reconstruction of the Graphene Monovacancy. *ACS Nano*, 7(5):4495–4502, May 2013.

[9] Jack D. Wadey, Alexander Markevich, Alex Robertson, Jamie Warner, Angus Kirkland, and Elena Besley. Mechanisms of monovacancy diffusion in graphene. *Chemical Physics Letters*, 648:161–165, March 2016.

[10] Jani Kotakoski, Clemens Mangler, and Jannik C. Meyer. Imaging atomic-level random walk of a point defect in graphene. *Nature Communications*, 5(1):3991, May 2014.

[11] Eva G. Noya, Itziar Zubieta, David J. Pine, and Francesco Sciortino. Assembly of clathrates from tetrahedral patchy colloids with narrow patches. *The Journal of Chemical Physics*, 151(9):094502, September 2019.

[12] Mingxin He, Johnathon P. Gales, Étienne Ducrot, Zhe Gong, Gi-Ra Yi, Stefano Sacanna, and David J. Pine. Colloidal diamond. *Nature*, 585(7826):524–529, September 2020.

[13] Andreas Neophytou, Dwaipayan Chakrabarti, and Francesco Sciortino. Facile self-assembly of colloidal diamond from tetrahedral patchy particles via ring selection. *Proceedings of the National Academy of Sciences*, 118(48):e2109776118, November 2021.

[14] Tianran Zhang, Dengping Lyu, Wei Xu, Yijiang Mu, and Yufeng Wang. Programming Self-Assembled Materials With DNA-Coated Colloids. *Frontiers in Physics*, 9, 2021.

[15] Jincan Zhang, Li Lin, Kaicheng Jia, Luzhao Sun, Hailin Peng, and Zhongfan Liu. Controlled Growth of Single-Crystal Graphene Films. *Advanced Materials*, 32(1):1903266, 2020.

[16] Mengqi Zeng, Lingxiang Wang, Jinxin Liu, Tao Zhang, Haifeng Xue, Yao Xiao, Zhihui Qin, and Lei Fu. Self-Assembly of Graphene Single Crystals with Uniform Size and Orientation: The First 2D Super-Ordered Structure. *Journal of the American Chemical Society*,

138(25):7812–7815, June 2016.

[17] P. Thevenaz, U.E. Ruttimann, and M. Unser. A pyramid approach to subpixel registration based on intensity. *IEEE Transactions on Image Processing*, 7(1):27–41, Jan./1998.

[18] Jay W. Grate, Marvin G. Warner, Jonathan W. Pittman, Karl J. Dehoff, Thomas W. Wietsma, Changyong Zhang, and Mart Oostrom. Silane modification of glass and silica surfaces to obtain equally oil-wet surfaces in glass-covered silicon micromodel applications. *Water Resources Research*, 49(8):4724–4729, 2013.

[19] Annina M. Steinbach, Tanja Sandner, Boris Mizaikoff, and Steffen Strehle. Gas phase silanization for silicon nanowire sensors and other lab-on-a-chip systems. *physica status solidi c*, 13(4):135–141, 2016.

[20] Walid-Madhat Munief, Florian Heib, Felix Hempel, Xiaoling Lu, Miriam Schwartz, Vivek Pachauri, Rolf Hempelmann, Michael Schmitt, and Sven Ingebrandt. Silane Deposition via Gas-Phase Evaporation and High-Resolution Surface Characterization of the Ultrathin Siloxane Coatings. *Langmuir*, 34(35):10217–10229, September 2018.
