# Supplementary material for: Visualizing defect dynamics by assembling the colloidal graphene lattice
Source: Nat Commun. 2023 Mar 18;14:1524. doi: 10.1038/s41467-023-37222-4 (PMC10024684; doi:10.1038/s41467-023-37222-4)
Supplement: Supplementary file 3 — Description of Additional Supplementary Files [file 41467_2023_37222_MOESM3_ESM.docx]

**Description of Additional Supplementary Files**

Supplementary Movie 1

Description: **Merging of two misaligned grains.** The movie shows reconfigurations at the grain boundary upon merging, eventually yielding a scar of penta- and heptagons. It is recorded during the assembly at *ΔT* = 0.10°C, and the images are taken with 63.5 second intervals.

Supplementary Movie 2

Description: **Healing of a colloidal graphene polycrystal**. The movie shows the long-time evolution of a polycrystal, corresponding to Figure 4 of the main text. Note the stark contrast between static and dynamic regions in the structure. The movie is recorded at *ΔT* = 0.05°C, and the images are taken with 184.8 second intervals.
